# Detection and Characterization of *Salmonella* Serotypes in the Production Chain of Two Pig Farms in Buenos Aires Province, Argentina

**DOI:** 10.3389/fmicb.2018.01370

**Published:** 2018-06-28

**Authors:** Rocío Colello, María J. Ruiz, Valeria M. Padín, Ariel D. Rogé, Gerardo Leotta, Nora Lía Padola, Analía I. Etcheverría

**Affiliations:** ^1^Laboratorio de Inmunoquímica y Biotecnología, Centro de Investigación Veterinaria de Tandil (CIVETAN), CONICET-CICPBA, Facultad de Ciencias Veterinarias, Universidad Nacional del Centro de la Provincia de Buenos Aires, Tandil, Argentina; ^2^Servicio Antígenos y Antisueros, Instituto Nacional de Producción de Biológicos, Administración Nacional de Laboratorios e Institutos de Salud “Dr. Carlos G. Malbrán”, Buenos Aires, Argentina; ^3^Instituto de Genética Veterinaria “Ing. Fernando Noel Dulout” (UNLP-CONICET LA PLATA), Facultad de Ciencias Veterinarias, Universidad Nacional de La Plata, Buenos Aires, Argentina

**Keywords:** *Salmonella* serotypes, prevalence, pork production chain, MDR, ERIC-PCR

## Abstract

The aim of the present study was to determine the prevalence of *Salmonella* in the pork production chain and to characterize *Salmonella* isolates. From 764 samples, 35 (4.6%) were positive for *Salmonella* spp., as determined by biochemical tests and the presence of the *invA* gene. From these, 2.6, 2.0, 8.8, and 8.0% corresponded to samples collected from farms, slaughterhouses, boning rooms and retail markets, respectively. *Salmonella* strains were classified into five serotypes and distributed as follows: *S.* Typhimurium in the pork production chain, *S*. Kentucky in farms and slaughterhouses, *S*. Brandenburg in slaughterhouses*, S*. Livingstone in farms and *S*. Agona in boning rooms and retail markets. Interestingly, the antimicrobial susceptibility testing indicated that all 35 *Salmonella* spp.-positive isolates were resistant to at least one antimicrobial agent, and 30 were multidrug-resistant (MDR) and resistant to different classes of antibiotics. The enterobacterial repetitive intergenic consensus-polymerase chain reaction (ERIC-PCR) analysis showed clonal relatedness among strains isolated from farms, boning rooms and retail markets. The presence of antibiotic-resistant *Salmonella* in food poses a potential health hazard to consumers.

## Introduction

*Salmonella* spp. are important zoonotic pathogens involved in human foodborne illness (Castagna et al., 2005; Sanchez-Maldonado et al., 2017). Most cases of salmonellosis are associated with ingestion of contaminated food such as poultry, milk, beef, pork, eggs, fruits and vegetables (Favier et al., 2013). Contaminated pork meat may be responsible for up to 25% of this illness, being *Salmonella* Typhimurium the most common serotype isolated (Boyen et al., 2008; Kich et al., 2011).

The reservoir of *Salmonella* is the intestinal tract of domestic animals, including pigs. *Salmonella* infection in pigs is sub-clinical; shedding is intermittent for long periods and leading the infection in some farms (Baggesen, 2006). The prevalence of shedding may increase from farm to slaughter because pigs are exposed to a variety of potential stressors during transport, increasing the number of animals carrying and shedding *Salmonella* as well as its levels in the gastrointestinal tract (Bonardi et al., 2013; Yang et al., 2017). Some slaughter operations, such as handling of the gastrointestinal tract, can influence the bacterial contamination of carcasses, equipment, floors and personnel (Bole-Hribovšek et al., 2008). In addition, environmental *Salmonella* serotypes could produce cross contamination on the slaughter line or during quartering. The molecular tracing of *Salmonella* isolates along the pork production chain represents a suitable tool to evaluate cross contamination (Hernández et al., 2013).

Molecular typing is a useful method for distinguishing among different bacterial isolates that can be used to trace the origins of pathogenic bacteria (Clark et al., 2012). For instance, the enterobacterial repetitive intergenic consensus-polymerase chain reaction (ERIC-PCR) analysis is useful to highlight relationships among strains of *Salmonella* isolated from different sources (Swanenburg et al., 1998; de Souza et al., 2015).

*Salmonella* gastroenteritis is a self-limiting illness although severe cases in immune-compromised, and elderly people or neonates may require effective antimicrobial therapy (White et al., 2001). The use of antimicrobial agents in human and veterinary medicine can lead to the emergence and spread of antimicrobial-resistant *Salmonella*, particularly multidrug-resistant (MDR) strains. Thus, infections with MDR *Salmonella* through contaminated food of animal origin have become a worldwide public health concern (Yang et al., 2017; Zhu et al., 2017).

In Argentina, the National Zoonotic Disease Control Program of the Ministry of Health has incorporated salmonellosis into more important zoonotic diseases of the country (Casas and Geffner, 2014). Nevertheless, few studies report the prevalence of *Salmonella* in the pork production chain in our country, so that the importance of this pathogen in the region is not well-established. Therefore, taking into account the hazard of consuming pork meat contaminated with *Salmonella* and the dissemination of MDR strains (Yang et al., 2017), the aim of this study was to determine the prevalence, serotypes and antibiotic resistance of *Salmonella* strains isolated in the pork production chain, and to assess the possible genetic relationships among *Salmonella* isolates by ERIC-PCR.

## Materials and Methods

### Management of Farms and Animals

The study was conducted in two pig farms which were intensively organized in total confinement. Production stages (gestation, farrowing, weaning and growing/finishing [fattening]) were geographically separated from each other within the same farm. When litters reached 70 days of age and a weight of 35 kg, they were transferred to the fattening or termination area. Each enclosure was divided into rooms and each room consisted of a variable number of pens depending on the size of the group. Partitions between pens were made of concrete. The usual group size varied between 10 and 30 pigs. Pigs and employees moved from one building to another by means of corridors isolated from external traffic. All herds received pelleted feed from the same manufacturer.

### Management of Carcasses Before Transport to Retail Markets

Pigs at the finishing production stage were transported to the slaughterhouse. After slaughtering, pork carcasses were chilled for 24–48 h and sent to boning rooms in refrigerated trucks, where they were boned to products such as meat and minced meat. Finally, the products were transferred to retail markets.

### Sample Collection

Seven hundred and sixty four samples were collected from two pig production systems, including farms, slaughterhouses, boning rooms and retail markets located in Buenos Aires province, Argentina, from 2012 to 2015.

This study was carried out in accordance with the recommendations of the Animal Welfare Committee from the School of Veterinary Sciences, UNCPBA, 087/02.

### Pig Farm Sampling

From a total of 348 samples collected, 277 corresponded to rectal swabs randomly taken from different animals at different production stages, and 71 were obtained from the farm environment by swabbing randomly drinking water, pelleted feed and feces on the floor.

### Slaughterhouse Sampling

A total of 147 samples were taken at slaughter. From these, 22 were from rectal swabs after slaughter, 85 from carcasses and 40 from the slaughter environment.

**FIGURE 1:**
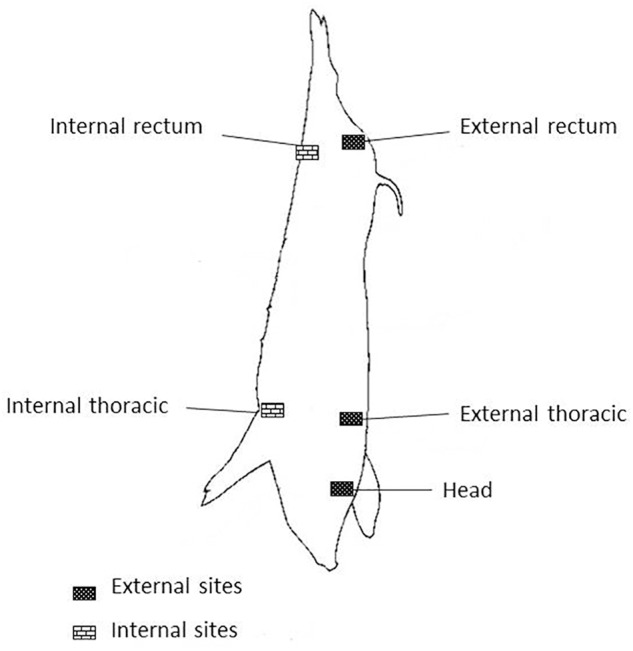
Sites on pig carcass.

Carcass swabs were taken according to memo No 3496/02 of the National Service of Agrifood Health and Quality (SENASA, for its Spanish acronym) (SENASA, 2002). Five quarter areas of 100 cm^2^ each were taken and processed separately (head, external rectum, internal rectum, external thoracic and internal thoracic) (**Figure 1**). Environmental samples were obtained at different points in the slaughter line (pre-washing, scalding, deharing, dressing and cooling) and from knives.

### Boning Room Sampling

From a total of 182 samples, 95 were collected from carcasses in the same way as slaughterhouse samples (**Figure 1**), 24 were from meat, 23 from minced meat and 40 from environmental samples obtained by swabbing randomly, refrigerated trucks and meat contact surfaces, such as meat tables, knives, meat mincing machine and vertical band saw machine.

### Retail Market Sampling

A total of 87 samples were collected by swabbing randomly meat (43), minced meat (13) and from the environment (31) namely, meat tables, knives, vertical band saw machines and refrigerators).

### Microbiological Analysis

Samples were processed according to the FDA Bacteriological Analytical Manual, with modifications. Briefly, each swab was homogenized in 225 ml of buffered peptone water and incubated at 37°C for 20 h. Then, 0.1 ml culture medium was inoculated into 10 ml Rappaport-Vassiliadis broth and incubated at 42°C for 24 h. Another 1 ml from the same pre-enrichment culture was inoculated into 10 ml of Tetrathionate Broth Base with iodine solution and incubated at 37°C for 24 h. Each selective enrichment broth was streaked onto Hektoen Enteric agar. Following incubation at 37°C for 24 h, presumptive *Salmonella* colonies were checked by Triple Sugar Iron (TSI) agar and Lysine Iron Agar (LIA).

### *invA* Gene Detection by PCR

All biochemically typical *Salmonella* isolates were analyzed by PCR to detect the *invA* gene (Rahn et al., 1992). DNA was extracted following methodologies previously described by Parma et al. (2000). Amplification of DNA was performed in a total volume of 50 μl. The reaction mixture contained 500 mM KCl, 100 mM Tris–HCl pH 9, Triton X-100, 25 mM MgCl_2_, 200 μM of each deoxynucleotide (dATP, dGTP, dCTP, dTTP), 1U *Taq*DNA Polymerase Highway^®^ (Inbio) and 5 μl DNA. The initial denaturation at 94°C for 10 min was followed by 30 cycles of denaturation at 94°C for 1 min, annealing at 60°C for 1 min and extension at 72°C for 2 min, with a final extension at 72°C for 10 min. Amplification products were separated by electrophoresis on 2% agarose gels containing 0.8 μg/ml ethidium bromide in running buffer and visualized by UV transillumination.

### Serotyping

*Salmonella* serotyping was performed according to the White–Kauffmann-Le Minor scheme by slide (O antigen) and tube (H antigen) agglutination, using specific antisera (Instituto Nacional de Producción de Biológicos (INPB) - ANLIS “Dr. Carlos G. Malbrán”, Argentina).

### Antibiotic Susceptibility

Isolate antibiotic susceptibility profiles were determined by the disk diffusion method according to the Clinical and Laboratory Standards Institute Guidelines (CLSI, 2014). The following antibiotics were assessed: ampicillin (AMP 10 ug), cephalothin (CEF 30 μg), cefotaxime (CTX 30 μg), cefoxitin (FOX 30 μg), amoxicillin/clavulanic acid (AMC 20/10 μg), gentamicin (GEN 10 μg), amikacin (AKN 30 μg), streptomycin (S 300 μg), tetracycline (TET 30 μg), nalidixic acid (NAL 30 μg), trimethoprim/sulfamethoxazole (TMS 1.25/23.75 μg), ciprofloxacin (CIP 5 μg), chloramphenicol (CMP 30 μg), nitrofurantoin (NIT 300 μg), fosfomycin (FOS 50 μg) and colistin (COL 10 μg). *Salmonella* isolates were reported as susceptible, intermediate or resistant (Famiglietti et al., 2005). Multidrug-resistance (MDR) was defined as strain resistance to three or more antibiotic families (Magiorakos et al., 2012).

### ERIC-PCR Analysis

All isolates were cultured in TSA (Britania), at 37°C for 24 h; four colonies were taken and then boiled for DNA extraction. The primers used for ERIC-PCR were: ERIC-1R (5′-ATGTAAGCTCCTGGGGATTCAC-3′) and ERIC-2 (5′-AAGTAAGTGACTGGGGTGAGCG-3′) (Versalovic et al., 1991). Amplification of DNA was performed in a total volume of 50 μl. The reaction mixture contained 500 mM KCL, 100 mM Tris–HCL pH 9, Triton X-100, 25 mM MgCl_2_, 200 μM of each deoxynucleotide (dATP, dGTP, dCTP, dTTP), 1U *Taq* DNA Polymerase from Highway^®^ and 5 μl DNA. The amplification cycles consisted in an initial denaturation at 94°C for 2 min, followed by 35 cycles of denaturation at 94°C for 30 s, primer annealing at 52°C for 1 min, an extension at 72°C for 4 min and a final extension at 74°C for 4 min. The amplification products were separated by 1.5% agarose gel electrophoresis using a 1 kb molecular weight marker plus ladder. Electrophoresis conditions were 100 V for 1 h in Tris-Borate-EDTA with ethyl bromide (0.8 μg/ml).

### Data Analysis

DNA fingerprints were analyzed by using a computer program for comparative analysis of DNA electrophoresis patterns (TotalLab Limited 2013). After normalization and alignment of the different DNA profiles, the relative genetic similarity among *Salmonella* spp. isolates was calculated and visualized by cluster analysis. ERIC-PCR products were defined as presence (a score of 1) and absence (a score of 0) of the DNA band. A dendrogram was generated with the BioNumerics vs. 6.6 software (Applied-Maths) using the Unweighted Pair Group Method with Arithmetic Mean (UPGMA). The discrimination index (*D*-value) was calculated by Simpson’s diversity index (Hunter and Gaston, 1988).

## Results

### Prevalence of *Salmonella* spp.

A total of 34 out of 764 samples (4.5%) were *Salmonella* spp.-positive, as determined by biochemical tests and presence of the *invA* gene.

In farms, 3% (11/348) of positive samples were found. From these, 0.7% (2/277) corresponded to the gestation stage and 9% (7/71) to the environment (pelleted feed and floor samples). In slaughterhouses, 2.0% (3/147) of samples were positive. All of them were isolated from the environment (holding pens, holding pen wastewater and rectum of a pig after slaughter). In boning rooms, 8.2% (15/182) of samples were *Salmonella* spp.-positive and namely from the environment (17.5%, 7/40), carcasses (6.3%, 6/95) and meat (4.2%, 2/47). The distribution of positive samples according to the different carcass quarters was 50% (3/6) from the external thoracic region, 16.6% (1/6) from the external rectum, 16.6% (1/6) from the internal rectum and 16.6% (1/6) from heads. In retail markets, 8.0% (7/87) of samples isolated from pork meat and minced meat ready for sale were positive for *Salmonella* spp.

### Serotyping

Strains were classified into five serotypes and distributed as follows: *Salmonella* Typhimurium along the pork production chain, *S*. Kentucky in farms and slaughterhouses, *S*. Brandenburg in slaughterhouses*, S*. Livingstone in farms and *S*. Agona in boning rooms and retail markets (**Table 1**). The prevalence of *S.* Typhimurium was significantly higher than that of other serotypes (77.2%), followed by *S*. Agona (11.4%), *S*. Kentucky (5.7%) *S*. Livingstone (2.9%) and *S*. Brandenburg (2.9%).

**Table 1:** Prevalence of *Salmonella* in farms, slaughterhouses, boning rooms and retail markets, and serotypes identified.

Source/sample isolate	Samples analyzed (n)	Positive samples (%)^(a)^	Serotype^(b)^
Farm			
Different categories	277	2 (0.7)	*S.* Typhimurium (2)
Environment	71	7 (9)	*S.* Typhimurium (5)
			*S*. Livingstone (1)
			*S.* Kentucky (1)
Slaughterhouse			
Rectal swabs	22	0 (0)	
Carcasses	85	0 (0)	
Environment	40	3 (7.5)	*S.* Typhimurium (1)
			*S*. Brandenburg (1)
			*S*. Kentucky (1)
Boning room			
Carcasses	95	6 (6.3)	*S.* Typhimurium (6)
Meat/minced meat	47	2 (4.2)	*S.* Typhimurium (2)
Environment	40	7 (17.5)	*S.* Typhimurium (5)
			*S*. Agona (2)
Retail market			
Meat	43	1 (2.3)	*S.* Typhimurium (1)
Minced meat	13	6 (46)	*S.* Typhimurium (5)
Environment	31	0 (0)	*S*. Agona (1)
Total samples	764	34 (4.5)	

*^(a)^Percentage of positive sample per type of sample. ^(b)^Number of serotypes isolated per sample.*

### Antibiotic Susceptibility of *Salmonella* Isolates

All of the 34 *Salmonella* isolates tested were resistant to at least one antimicrobial agent, whereas 30 were MDR and resistant to different classes of antibiotics, including β-lactamase, fluoroquinolones, chloramphenicol, aminoglycosides and tetracyclines. Resistance to β-lactams ampicillin (86.1%) and amoxicillin/clavulanic acid (19.4%) was most frequently observed, followed by cephalothin and cefoxitin (16.6%). Percentages of resistance to aminoglycosides such as gentamicin, streptomycin and amikacin were 86, 5.5, and 5.6%, respectively. In the case of tetracycline, 80.5% of isolates showed resistance. Concerning fluoroquinolones, percent resistance was 72.2 and 8.3% to nalidixic acid and ciprofloxacin, respectively. *Salmonella* isolates also exhibited resistance to chloramphenicol (22%), colistin (8.8%) and fosfomycin (2.8%). Some isolates exhibited intermediate sensitivity to cephalothin (38.8%), amoxicillin/clavulanic acid (36.6%) and colistin (27.7%). When antimicrobial resistance was analyzed by source of isolates, farms and retail markets showed the highest rate of resistance to antibiotics of all classes, followed by boning rooms and slaughterhouses. When analyzed by serotype, *S.* Typhimurium and *S*. Agona were the most resistant, followed by *S*. Brandenburg, *S*. Kentucky and *S*. Livingstone (**Table 2**).

**Table 2:** Antimicrobial resistance of *Salmonella* according to source and serotype.

Source/sample isolate	Serotype	Resistance (%)															

		AMP	CEF	CTX	FOX	A/CL	GEN	AKN	S	TET	NAL	TMS	CIP	CMP	NIT	FOS	COL
Farm																	
Different categories	*S.* Typhimurium (2)	100	0	0	50	50	100	50	0	100	100	0	50	50	0	0	50
Environment	*S.* Typhimurium (5)	100	0	20	20	0	100	0	0	100	100	0	20	20	0	0	0
	*S*. Livingstone (1)	0	0	0	0	0	0	0	0	0	0	0	0	100	0	0	0
	*S.* Kentucky (1)	100	0	0	0	0	0	0	0	0	0	0	0	0	0	0	0
Slaughterhouse																	
Rectal swabs	*S.* Typhimurium (1)	100	0	0	0	0	0	0	0	100	0	0	0	0	0	0	0
Carcasses	*S*. Brandenburg (1)	100	0	0	0	0	0	0	0	0	0	0	0	100	0	0	0
Environment	*S*. Kentucky (1)	0	0	0	0	0	0	0	0	100	0	0	0	0	0	0	0
Boning room																	
Carcasses	*S.* Typhimurium (6)	100	33	0	33	16	100	0	0	100	100	16	0	16	0	0	0
Meat/minced meat	*S.* Typhimurium (2)	100	0	0	50	0	100	0	0	100	0	0	0	0	0	0	0
Environment	*S.* Typhimurium (5)	80	20	20	0		80	0	0	80	80	0	0	0	0	0	0
	*S*. Agona (2)	0	50	0	0	50	100	50	0	0	0	0	0	50	50	0	0
Retail market																	
Meat	*S.* Typhimurium (1)	100	33	0	0	66	100	0	16	100	100	0	0	16	0	16	0
Minced meat	*S.* Typhimurium (5)	100	0	0	0	0	0	100	0	100	100	0	0	0	0	0	0
	*S*. Agona (1)	0	0	0	100	100	100	100	100	0	0	0	0	0	0	0	100

### Subtyping by ERIC-PCR

**FIGURE 2:**
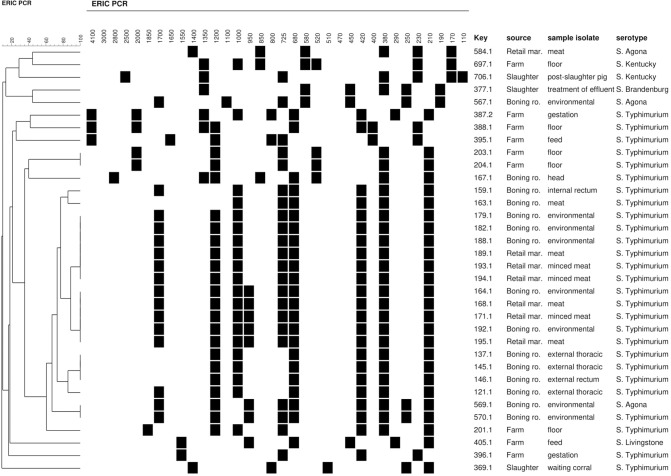
Dendrogram showing genetic relatedness, source, sample type and serotype of *Salmonella* strains isolated along the pork production chain. (^∗^) Multiple DNA fragments of all strains generated with ERIC-PCR are shown on top of the figure. Black, presence of band; White, absence of band.

All *Salmonella* spp. strains were analyzed by ERIC-PCR. The relationships among isolates on the basis of ERIC fingerprints are presented in **Figure 2**. Multiple DNA fragments of all strains generated with ERIC primers were composed of 6–10 bands ranging between 100 bp and 4 Kb.

The ERIC-PCR analysis and strain clustering produced 18 strains grouped in five clusters (I and V) and 16 strains with unique patterns at a *D*-value of 0.90. Strains with identical profile were isolated from different sources. Strains of clusters II, III, IV, and V presented the same serotype, strains were isolated from boning room (I and IV), farm (V) and boning room and retail markets (II and III). Cluster I had two different serotypes (*S*. Typhimurium (Identical *S*. Typhimurium and *S*. Agona) and two strains isolated from the boning room environment. Cluster II included five strains, two isolated from boning rooms (carcasses and environment) and three isolated from retail markets (meat). Cluster III included three strains from the boning room environment and other three from retail markets (meat and minced meat). Cluster IV comprised three strains from the boning room environment and cluster V contained two strains isolated from farm floor.

## Discussion

In this study, the characterization of *Salmonella* strains in the pork production chain is reported. The prevalence of *Salmonella* spp. at different stage of production from other countries are variable and it is important when comparing prevalence since the variation may be due to factors, such as sampling method and samples processing.

Although the routes of access of *Salmonella* onto pork meat differ according to the stage of the process, the main factor is the supply of *Salmonella* colonized pigs onto the slaughter line, with the consequent contamination of carcasses and meat, both sources of foodborne pathogens (Kirchner et al., 2011). Evisceration and subsequent cross-contamination of neighboring carcasses by splash, handling and contact with surfaces are all important aspects (Bole-Hribovšek et al., 2008). In our study, *Salmonella* was detected in 3% of pig farm samples, as opposed to the high prevalence reported by Kich et al. (2011) in Brazil and Bonardi et al. (2013) in Italy. The main factor of pig farm *Salmonella* epidemiology is concerned with the introduction of bacteria, the subsequent transmission to pigs and the introduction of contaminated feed (Vigo et al., 2009). We found positive samples in feed, as reported by Wong et al. (2002), who reported that feed can be considered a risk factor for *Salmonella*.

Our results showed that the prevalence of *Salmonella* in slaughterhouses, boning rooms and retail markets was 2.0, 8.2 and 8.0%, respectively. The information gathered from carcasses in boning rooms and meat from retail markets agreed with that reported in previous studies from different countries showing the high prevalence of *Salmonella* in pig carcasses and meat. For instance, the prevalence of *Salmonella* was 16.7% in China (Li et al., 2013), 10.86% in Spain (Hernández et al., 2013), 13.8% in Germany (Mihaiu et al., 2014) and 24.1% in Argentina (Ibar et al., 2009). Since some carcass areas are more likely exposed to potential contamination or cross contamination, sampling at three or four carcass sites is recommended. The external area involves a particular risk of contamination during the early stages of dressing (Roberts et al., 1984). Our findings showed that the external surface was the most contaminated area, whereas the prevalence of *Salmonella* in equipment was 17.5%, including splitting and mixing machines, processing table and hook. The rol of equipment in carcasses contamination is important, partly due to the possible buildup of bacteria in or on the equipment during working hours (Wong et al., 2002). In retail markets, *Salmonella* recovery was 8.04%, higher than the 0.3 and 4.3% reported by Delhalle et al. (2009) in different pork retailers in Belgium. Contamination levels from pork meat in retail markets depend mainly on the quality of raw materials and products, handling, time and temperature.

All isolates analyzed were genetically confirmed as *Salmonella*-positive by the presence of the *invA* gene. This result is in agreement with that previously reported by other authors (Oliveira et al., 2002; Kumar et al., 2009).

One of the most common serotypes causing human salmonellosis in many countries is *S*. Typhimurium (Campos et al., 2012; Sanchez-Maldonado et al., 2017), which was the main serotype identified in this study. In other reports, this serotype was also found to be predominant in pig and pork products (Botteldoorn et al., 2003; Kich et al., 2011; Bonardi et al., 2013), while other serotypes such as *S*. Agona, *S*. Brandenburg, *S*. Kentucky and *S*. Livingstone were also reported in pigs in previous studies (Botteldoorn et al., 2003; Hernández et al., 2013).

The surveillance of *Salmonella* resistant to antimicrobial vary from 20 to 30% in the 1990s to 70% in some countries in 2000s (Su et al., 2004). The use of antimicrobials in food animals as growth promoters and metaphylactic, prophylactic and therapeutic agents, allows the emergence of antimicrobial-resistant *Salmonella* (Yang et al., 2010). Our findings are similar to those previously described, showing that *Salmonella* isolates from pigs and pork meat are commonly MDR. However, resistance rate was much higher than that reported in the United States and China (Chen et al., 2004), Romania (Mihaiu et al., 2014) and Argentina (Ibar et al., 2009), and the highest frequency was for ampicillin resistance, followed by gentamicin, tetracycline and nalidixic acid. Similar results were found in *Salmonella* isolates from other countries (Thakur et al., 2007; Kich et al., 2011). Fluoroquinolones and cephalosporins are potentially lifesaving treatments for extraintestinal infections. Interestingly, the co-resistance to fluoroquinolones and cephalosporins found in our isolates could limit the effective treatment of *Salmonella* infections in humans, as reported by Li et al. (2013). Colistin is an antimicrobial peptide commercialized in both human and veterinary medicine which has been extensively used orally in pigs for the control of *Enterobacteriaceae* infections (Olaitan et al., 2014; Rebelo et al., 2018). In the present study, 8.8% of strains were colistin-resistant, suggesting the possible loss of colistin effectiveness in human treatment. In addition, is necessary the establishment of a guidelines for the use of colistin in pigs in countries where this drug is approved (Rhouma et al., 2016).

Of the serotypes identified in the present study, *S.* Typhimurium and *S*. Agona showed the highest rates of antimicrobial resistance and MDR. On the other hand, serotypes Kentucky, Livingstone and Brandenburg were relatively more susceptible to antimicrobial agents, indicating that the spread of MDR *S.* Typhimurium isolates is potentially serious, as already reported (Yang et al., 2010; Li et al., 2013).

Swanenburg et al. (1998) standardized ERIC-PCR, a very useful method for quick typing of many *Salmonella* isolates. ERIC analysis showed clonal relatedness among strains isolated from boning rooms and retail markets, probably due to cross contamination in the deboning process. Five clusters grouped clonal *Salmonella* strains obtained from at least two types of samples. This method is simple, rapid and cheap for typing bacterial strains associated with foodborne outbreaks (Adzitey et al., 2013). However, we could not differentiate even intra-serotype isolates, as reported by Fendri et al. (2013). Future analyses using reliable techniques for discriminating different *Salmonella* serotypes, such as pulsed-field gel electrophoresis, could be appropriate.

Based on our results, isolates from different sources may have originated from a single clone and transmitted along the production chain. That cross-contamination has considerable potential of further spread and dissemination of *Salmonella* spp.

## Conclusion

Our findings demonstrate the occurrence of *Salmonella* contamination along the pork production chain in Buenos Aires province, Argentina. Surveillance of *Salmonella* in pork meat and characterization of isolates can contribute to the understanding of the epidemiology of this pathogen. Additionally, many *Salmonella* isolates were resistant to multiple antimicrobials, and the presence of this pathogen in the food chain represents a risk for human health. The high rates of MDR *Salmonella* detected suggest that some measures should be taken for the reasonable use of antimicrobials in animal husbandry. These results reinforce the need of an integrated *Salmonella* control program based on pre-harvest good management practices in the farm. A prudent use of antimicrobials and control of critical point systems at post-harvest should be implemented to decrease the hazard of *Salmonella* transmission to consumers. Therefore, implementation of proper hygiene practices during the pork meat production process should be enforced. Isolate characterization should contribute to the understanding of *Salmonella* epidemiology and to conducting food surveillance directed toward this pathogen. Implementation of a comprehensive program covering the entire food value chain continuum from ‘farm to fork’ is important for *Salmonella* control.

## Author Contributions

RC conceived, designed, analyzed the experiments, and wrote the manuscript. MR, VP, and AR did some of the experiments. GL, NP, and AE designed some of the experiments, analyzed the data, and revised the manuscript.

## Conflict of Interest Statement

The authors declare that the research was conducted in the absence of any commercial or financial relationships that could be construed as a potential conflict of interest.

## References

[B1] Adzitey F. Ali G. R. R. Huda N. Cogan T. Corry J. (2013). Prevalence, antibiotic resistance and genetic diversity of *Listeria monocytogenes* isolated from ducks, their rearing and processing environments in Penang, Malaysia. *Food Control* 32 607–614. 10.1016/j.foodcont.2012.12.016

[B2] Baggesen D. L. (2006). *Opinion of the Scientific Panel on Biological Hazards on the Request from the Commission Related to “Risk Assessment and Mitigation Options of Salmonella in Pig Production.* Question No EFSA-Q-2005–2019. Parma: European Food Safety Authority.

[B3] Bole-Hribovšek V. Chriél M. Davies R. Fanning J. van de Giessen A. W. Palancar L. P. (2008). *Salmonella in Holdings with Breeding Pigs in the EU, 2008: Part A: Salmonella Prevalence Estimates.* Parma: European Food Safety Authority.

[B4] Bonardi S. Bassi L. Brindani F. D’Incau M. Barco L. Carra E. (2013). Prevalence, characterization and antimicrobial susceptibility of *Salmonella enterica* and *Yersinia enterocolitica* in pigs at slaughter in Italy. *Int. J. Food Microbiol.* 163 248–257. 10.1016/j.ijfoodmicro.2013.02.012 23603278

[B5] Botteldoorn N. Heyndrickx M. Rijpens N. Herman L. (2003). Detection and characterization of verotoxigenic *Escherichia coli* by a VTEC/EHEC multiplex PCR in porcine faeces and pig carcass swabs. *Res. Microbiol.* 154 97–104. 10.1016/S0923-2508(03)00028-7 12648724

[B6] Boyen F. Haesebrouck F. Maes D. Van Immerseel F. Ducatelle R. Pasmans F. (2008). Non-typhoidal *Salmonella* infections in pigs: a closer look at epidemiology, pathogenesis and control. *Vet. Microbiol.* 130 1–19. 10.1016/j.vetmic.2007.12.017 18243591

[B7] Campos J. Pichel M. Vaz T. Tavechio A. Fernandes S. Muñoz N. (2012). Building PulseNet Latin America and Caribbean *Salmonella* regional database: first conclusions of genetic subtypes of *S*. Typhi, *S*. Typhimurium and *S*. Enteritidis circulating in six countries of the region. *Food Res. Int.* 45 1030–1036. 10.1016/j.foodres.2011.10.020

[B8] Casas N. Geffner L. (2014). *National Control Programme of Zoonotic Diseases: Surveillance, Prevention and Control of Foodborne Zoonotic Pathogens [Online].* Available at: www.aazoonosis.org.ar/congreso/web/uploads/resumenes/Casas.doc (accessed May 2018).

[B9] Castagna S. M. F. Muller M. Macagnan M. Rodenbusch C. R. Canal C. W. Cardoso M. (2005). Detection of *Salmonella* sp. from porcine origin: a comparison between a PCR method and standard microbiological techniques. *Braz. J. Microbiol.* 36 373–377. 10.1590/S1517-83822005000400013

[B10] Chen S. Zhao S. White D. G. Schroeder C. M. Lu R. Yang H. (2004). Characterization of multiple-antimicrobial-resistant *Salmonella* serovars isolated from retail meats. *Appl. Environ. Microbiol.* 70 1–7. 10.1128/AEM.70.1.1-7.2004 14711619 PMC321239

[B11] Clark C. G. Taboada E. Grant C. C. Blakeston C. Pollari F. Marshall B. (2012). Comparison of molecular typing methods useful for detecting clusters of *Campylobacter jejuni* and *C. coli* isolates through routine surveillance. *J. Clin. Microbiol.* 50 798–809. 10.1128/JCM.05733-11 22162562 PMC3295161

[B12] CLSI (2014). *M100-S24 Performance Standards for Antimicrobial Susceptibility Testing, 24th Informational Supplement*, 24 Edn Wayne, PA: Clinical and Laboratory Standards Institute.

[B13] de Souza A. I. de Freitas Neto O. C. Batista D. F. Estupinan A. L. de Almeida A. M. Barrow P. A. (2015). ERIC-PCR genotyping of field isolates of *Salmonella enterica* subsp. *enterica* serovar *Gallinarum* biovars *gallinarum* and *pullorum*. *Avian Pathol.* 44 475–479. 10.1080/03079457.2015.1086975 26365161

[B14] Delhalle L. Saegerman C. Farnir F. Korsak N. Maes D. Messens W. (2009). *Salmonella* surveillance and control at post-harvest in the Belgian pork meat chain. *Food Microbiol.* 26 265–271. 10.1016/j.fm.2008.12.009 19269567

[B15] Famiglietti A. Quinteros M. Vázquez M. Marín M. Nicola F. Radice M. (2005). Consenso sobre las pruebas de sensibilidad a los antimicrobianos en *Enterobacteriaceae*. *Rev. Argent. Microbiol.* 37 57–66. 15991480

[B16] Favier G. I. Estrada C. S. L. Otero V. L. Escudero M. E. (2013). Prevalence, antimicrobial susceptibility, and molecular characterization by PCR and pulsed field gel electrophoresis (PFGE) of *Salmonella* spp. isolated from foods of animal origin in San Luis, Argentina. *Food Control* 29 49–54. 10.1016/j.foodcont.2012.05.056

[B17] Fendri I. Hassena A. B. Grosset N. Barkallah M. Khannous L. Chuat V. (2013). Genetic diversity of food-isolated *Salmonella* strains through pulsed field gel electrophoresis (PFGE) and Enterobacterial repetitive intergenic consensus (ERIC-PCR). *PloS One* 8:e81315. 10.1371/journal.pone.0081315 24312546 PMC3849149

[B18] Hernández M. Gómez-Laguna J. Luque I. Herrera-León S. Maldonado A. Reguillo L. (2013). *Salmonella* prevalence and characterization in a free-range pig processing plant: tracking in trucks, lairage, slaughter line and quartering. *Int. J. Food Microbiol.* 162 48–54. 10.1016/j.ijfoodmicro.2012.12.026 23353554

[B19] Hunter P. R. Gaston M. A. (1988). Numerical index of the discriminatory ability of typing systems: an application of Simpson’s index of diversity. *J. Clin. Microbiol.* 26 2465–2466. 3069867 10.1128/jcm.26.11.2465-2466.1988 PMC266921

[B20] Ibar M. Vigo G. Piñeyro P. Caffer M. Quiroga P. Perfumo C. (2009). Serovariedades de *Salmonella enterica* subespecie *enterica* en porcinos de faena y su resistencia a los antimicrobianos. *Rev. Argent. Microbiol.* 41 156–162. 19831314

[B21] Kich J. D. Coldebella A. Morés N. Nogueira M. G. Cardoso M. Fratamico P. M. (2011). Prevalence, distribution, and molecular characterization of *Salmonella* recovered from swine finishing herds and a slaughter facility in Santa Catarina, Brazil. *Int. J. Food Microbiol.* 151 307–313. 10.1016/j.ijfoodmicro.2011.09.024 22024043

[B22] Kirchner M. Marier E. Miller A. Snow L. McLaren I. Davies R. (2011). Application of variable number of tandem repeat analysis to track *Salmonella enterica* ssp. *enterica* serovar Typhimurium infection of pigs reared on three British farms through the production cycle to the abattoir. *J. Appl. Microbiol.* 111 960–970. 10.1016/j.ijfoodmicro.2011.09.024 21722278

[B23] Kumar R. Surendran P. Thampuran N. (2009). Distribution and genotypic characterization of *Salmonella* serovars isolated from tropical seafood of Cochin, India. *J. Appl. Microbiol.* 106 515–524. 10.1111/j.1365-2672.2008.04020.x 19200318

[B24] Li R. Lai J. Wang Y. Liu S. Li Y. Liu K. (2013). Prevalence and characterization of *Salmonella* species isolated from pigs, ducks and chickens in Sichuan Province, China. *Int. J. Food Microbiol.* 163 14–18. 10.1016/j.ijfoodmicro.2013.01.020 23474653

[B25] Magiorakos A. P. Srinivasan A. Carey R. Carmeli Y. Falagas M. Giske C. (2012). Multidrug-resistant, extensively drug-resistant and pandrug-resistant bacteria: an international expert proposal for interim standard definitions for acquired resistance. *Clin. Microbiol. Infect.* 18 268–281. 10.1111/j.1469-0691.2011.03570.x 21793988

[B26] Mihaiu L. Lapusan A. Tanasuica R. Sobolu R. Mihaiu R. Oniga O. (2014). First study of *Salmonella* in meat in Romania. *J. Infect. Dev. Ctries* 8 50–58. 10.3855/jidc.3715 24423712

[B27] Olaitan A. O. Morand S. Rolain J.-M. (2014). Mechanisms of polymyxin resistance: acquired and intrinsic resistance in bacteria. *Front. Microbiol.* 5:643 10.3389/fmicb.2014.00643 PMC4244539 25505462

[B28] Oliveira S. Santos L. Schuch D. Silva A. Salle C. Canal C. (2002). Detection and identification of salmonellas from poultry-related samples by PCR. *Vet. Microbiol.* 87 25–35. 10.1016/S0378-1135(02)00028-7 12079744

[B29] Parma A. Sanz M. Blanco J. Blanco J. Viñas M. Blanco M. (2000). Virulence genotypes and serotypes of verotoxigenic *Escherichia coli* isolated from cattle and foods in Argentina. *Eur. J. Epidemiol.* 16 757–762. 10.1023/A:1026746016896 11142505

[B30] Rahn K. De Grandis S. Clarke R. McEwen S. Galan J. Ginocchio C. (1992). Amplification of an *invA* gene sequence of *Salmonella* Typhimurium by polymerase chain reaction as a specific method of detection of *Salmonella*. *Mol. Cell. Probes* 6 271–279. 10.1016/0890-8508(92)90002-F 1528198

[B31] Rebelo A. R. Bortolaia V. Kjeldgaard J. S. Pedersen S. K. Leekitcharoenphon P. Hansen I. M. (2018). Multiplex PCR for detection of plasmid-mediated colistin resistance determinants, *mcr-1, mcr-2, mcr-3, mcr-4* and *mcr-5* for surveillance purposes. *Euro Surveill.* 23 17-00672. 10.2807/1560-7917.ES.2018.23.6.17-00672 29439754 PMC5824125

[B32] Rhouma M. Beaudry F. Thériault W. Letellier A. (2016). Colistin in pig production: chemistry, mechanism of antibacterial action, microbial resistance emergence, and one health perspectives. *Front. Microbiol.* 7:1789. 10.3389/fmicb.2016.01789 27891118 PMC5104958

[B33] Roberts T. Hudson W. Whelehan O. Simonsen B. Olgaard K. Labots H. (1984). Number and distribution of bacteria on some beef carcasses at selected abattoirs in some member states of the European Communities. *Meat Sci.* 11 191–205. 10.1016/0309-1740(84)90037-8 22054857

[B34] Sanchez-Maldonado A. F. Aslam M. Service C. Narváez-Bravo C. Avery B. P. Johnson R. (2017). Prevalence and antimicrobial resistance of *Salmonella* isolated from two pork processing plants in Alberta, Canada. *Int. J. Food Microbiol.* 241 49–59. 10.1016/j.ijfoodmicro.2016.10.004 27750110

[B35] SENASA (2002). *Circular 3496/02.* Martinez, CA: Servicio Nacional de Sanidad y Calidad Agroalimentaria.

[B36] Su L.-H. Chiu C.-H. Chu C. Ou J. T. (2004). Antimicrobial resistance in nontyphoid *Salmonella* serotypes: a global challenge. *Clin. Infect. Dis.* 39 546–551. 10.1086/422726 15356819

[B37] Swanenburg M. Urlings H. Keuzenkamp D. Snijders J. (1998). Validation of ERIC PCR as a tool in epidemiologic research of *Salmonella* in slaughter pigs. *J. Ind. Microbiol. Biotechnol.* 21 141–144. 10.1086/422726 15356819

[B38] Thakur S. Tadesse D. A. Morrow M. Gebreyes W. A. (2007). Occurrence of multidrug resistant *Salmonella* in antimicrobial-free (ABF) swine production systems. *Vet. Microbiol.* 125 362–367. 10.1016/j.vetmic.2007.05.025 17644277

[B39] Versalovic J. Koeuth T. Lupski R. (1991). Distribution of repetitive DNA sequences in eubacteria and application to finerpriting of bacterial enomes. *Nucleic Acids Res.* 19 6823–6831. 10.1093/nar/19.24.6823 1762913 PMC329316

[B40] Vigo G. B. Cappuccio J. A. Pineyro P. E. Salve A. Machuca M. A. Quiroga M. A. (2009). *Salmonella enterica* subclinical infection: bacteriological, serological, pulsed-field gel electrophoresis, and antimicrobial resistance profiles—longitudinal study in a three-site farrow-to-finish farm. *Foodborne Pathog. Dis.* 6 965–972. 10.1089/fpd.2008.0239 19642916 PMC3145166

[B41] White D. G. Zhao S. Sudler R. Ayers S. Friedman S. Chen S. (2001). The isolation of antibiotic-resistant *Salmonella* from retail ground meats. *N. Engl. J. Med.* 345 1147–1154. 10.1056/NEJMoa010315 11642230

[B42] Wong D. L. F. Hald T. Van Der Wolf P. Swanenburg M. (2002). Epidemiology and control measures for *Salmonella* in pigs and pork. *Livest. Prod. Sci.* 76 215–222. 10.1016/S0301-6226(02)00121-5

[B43] Yang B. Qu D. Zhang X. Shen J. Cui S. Shi Y. (2010). Prevalence and characterization of *Salmonella* serovars in retail meats of marketplace in Shaanxi, China. *Int. J. Food Microbiol.* 141 63–72. 10.1016/j.ijfoodmicro.2010.04.015 20493570

[B44] Yang L. Li W. Jiang G.-Z. Zhang W.-H. Ding H.-Z. Liu Y.-H. (2017). Characterization of a P1-like bacteriophage carrying CTX-M-27 in *Salmonella* spp. resistant to third generation cephalosporins isolated from pork in China. *Sci. Rep.* 7:40710. 10.1038/srep40710 28098241 PMC5241659

[B45] Zhu Y. Lai H. Zou L. Yin S. Wang C. Han X. (2017). Antimicrobial resistance and resistance genes in *Salmonella* strains isolated from broiler chickens along the slaughtering process in China. *Int. J. Food Microbiol.* 259 43–51. 10.1016/j.ijfoodmicro.2017.07.023 28800411

